# Isoquercitrin Inhibits Hydrogen Peroxide-Induced Apoptosis of EA.hy926 Cells via the PI3K/Akt/GSK3β Signaling Pathway

**DOI:** 10.3390/molecules21030356

**Published:** 2016-03-16

**Authors:** Meixia Zhu, Jiankuan Li, Ke Wang, Xuliang Hao, Rui Ge, Qingshan Li

**Affiliations:** 1 Department of Pharmacy Shanxi, College of Traditional Chinese Medicine, No. 121 University Avenue, Taiyuan 030619, China; meixiaz032@163.com (M.Z.); wangk1758@163.com (K.W.); 2 Shanxi Province Academy of Traditional Chinese Medicine, No. 46 Bingzhou West Road, Taiyuan 030012, China; 3 School of Pharmaceutical Science, Shanxi Medical University, No. 56 Xinjian South Road, Taiyuan 030001, China; lijiankuan2004@163.com (J.L.); cpugr@126.com (R.G.)

**Keywords:** isoquercitrin, EA.hy926 cells, apoptosis, oxidative stress, PI3K/Akt/GSK3β

## Abstract

Oxidative stress plays a critical role in endothelial injury and the pathogenesis of diverse cardiovascular diseases, including atherosclerosis. Isoquercitrin (quercetin-3-glucoside), a flavonoid distributed widely in plants, exhibits many biological activities, including anti-allergic, anti-viral, anti-inflammatory, and anti-oxidative effects. In the present study, the inhibitory effect of isoquercitrin on H_2_O_2_-induced apoptosis of EA.hy926 cells was evaluated. MTT assays showed that isoquercitrin significantly inhibited H_2_O_2_-induced loss of viability in EA.hy926 cells. Hoechst33342/PI and Annexin V-FITC/PI fluorescent double staining indicated that isoquercitrin inhibited H_2_O_2_-induced apoptosis of EA.hy926 cells. Western blotting demonstrated that isoquercitrin prevented H_2_O_2_-induced increases in cleaved caspase-9 and cleaved caspase-3 expression, while increasing expression of anti-apoptotic protein Mcl-1. Additionally, isoquercitrin significantly increased the expression of p-Akt and p-GSK3β in a dose-dependent manner in EA.hy926 cells. LY294002, a PI3K/Akt inhibitor, inhibited isoquercitrin-induced GSK3β phosphorylation and increase of Mcl-1 expression, which indicated that regulation of isoquercitrin on Mcl-1 expression was likely related to the modulation of Akt activation. These results demonstrated that the anti-apoptotic effect of isoquercitrin on H_2_O_2_-induced EA.hy926 cells was likely associated with the regulation of isoquercitrin on Akt/GSK3β signaling pathway and that isoquercitrin could be used clinically to interfere with the progression of endothelial injury-associated cardiovascular disease.

## 1. Introduction

Cardiovascular disease is a primary cause of morbidity and mortality worldwide. It is estimated that the annual number of deaths due to cardiovascular disease will increase from 17 million in 2008 to 25 million in 2030 [1]. The endothelium is the inner layer of the vasculature, which represents the interface between blood and organ systems, secretes numerous vasoactive substances, and regulates blood flow [2,3]. A large body of research has shown that endothelial dysfunction is related to the pathogenesis of cardiovascular diseases such as atherosclerosis, peripheral artery disease, and glomerulosclerosis [4,5,6]. Reactive oxygen species (ROS) generated excessively in the endothelium, including H_2_O_2_, HO·, and O_2_^−^, are major mediators responsible for oxidative stress which results in endothelial dysfunction [7,8,9]. Therefore, to protect endothelial cells from ROS-induced apoptosis has proven to be an effective intervention against the progress of some cardiovascular diseases such as atherosclerosis.

Isoquercitrin, also known as quercetin-3-glucoside, is a glucose-bound derivative of quercetin that is commonly found in medicinal herbs, fruits, vegetables, and plant-derived foods and beverages [10,11,12,13,14]. In recent studies, isoquercitrin has been reported to possess anti-Mayaro virus, anti-inflammatory, anti-oxidative, anti-allergic, anti-hypertensive, anti-hyperglycemic, and saluretic/diuretic effects [15,16,17,18,19,20,21,22,23]. Although the antioxidative and neuroprotective effects of isoquercitrin have been established, whether isoquercitrin could exert an anti-apoptotic effect on endothelial cells is not clear. Thus, the present study was designed to evaluate the effect of isoquercitrin on H_2_O_2_-induced EA.hy926 cell apoptosis.

## 2. Results

### 2.1. Isoquercitrin Inhibits H_2_O_2_-Induced Loss of Cell Viability in EA.hy926 Cells

**Figure 1:**
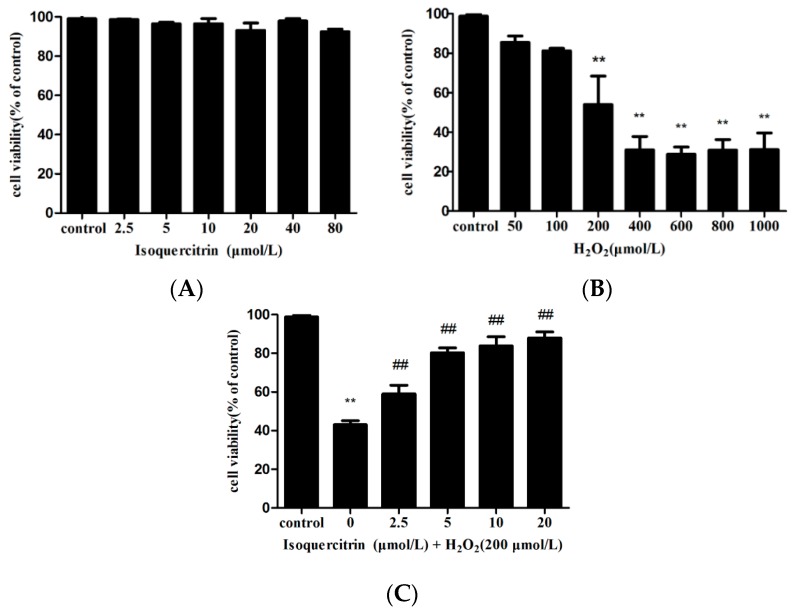
Protective activity of isoquercitrin measured by the MTT assay. (**A**) Cell viability of EA.hy926 cells treated with different concentrations of isoquercitrin; (**B**) Cell viability of EA.hy926 cells treated with different concentrations of H_2_O_2_; (**C**) Cell viability of EA.hy926 cells treated with isoquercitrin followed by H_2_O_2_ treatment. ** *p* < 0.01 *vs.* control; ^##^ *p* < 0.01 *vs.* H_2_O_2_ group.

The protective effect of isoquercitrin against H_2_O_2_-induced loss of viability in EA.hy926 cells was measured using the MTT assay. Cell viability was not significantly affected by exposure to 2.5–80 µmol/L isoquercitrin and decreased by 54.08% ± 7.02% after exposure to 200 μmol/L H_2_O_2_ for 4 h (Figure 1A,B). As illustrated in Figure 1C, the cells treated with 20 µmol/L isoquercitrin for 24 h before the addition of H_2_O_2_ significantly attenuated the viability loss induced by H_2_O_2_ (*p* < 0.01). These findings suggest that isoquercitrin inhibits H_2_O_2_-induced EA.hy926 cell death.

### 2.2. Isoquercitrin Inhibits H_2_O_2_-Induced Apoptosis of EA.hy926 Cells

**Figure 2:**
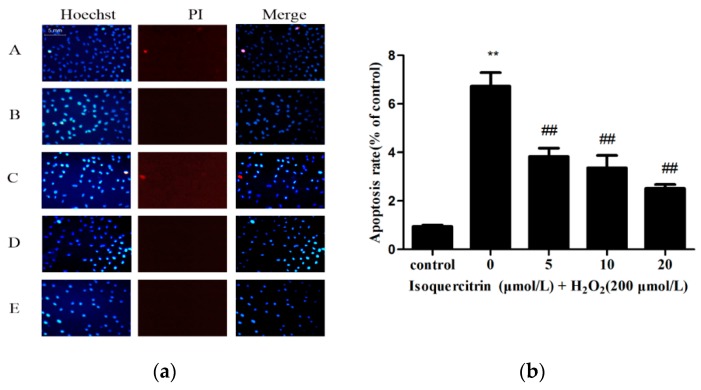
Hoechst 33342 and PI staining in EA.hy926 cells. (**a**) Representative fluorescence images obtained after Hoechst 33342/PI staining (A) Control group; (B) H_2_O_2_ treatment group; (C–E) 5, 10, and 20 μmol/L isoquercitrin, respectively, followed by the treatment of 200 μmol/L H_2_O_2_; (**b**) Percentages of apoptotic cells in total EA.hy926 cells. ** *p* < 0.01 *vs.* control. ^##^ *p* < 0.01 *vs.* H_2_O_2_ treatment group.

Hoechst 33342 dye causes bright blue fluorescence in apoptotic cells because of the high permeability of cell membranes, while propidium iodide (PI) dye causes red fluorescence in the nuclei of dead cells. Hoechst 33342/PI double staining was performed to compare the apoptotic rates of EA.hy926 cells treated with H_2_O_2_ alone and those treated with H_2_O_2_ and isoquercitrin (Figure 2). The apoptotic rate of the control group was 7.16% ± 1.18%, whereas that of the H_2_O_2_-treated group was 47.09% ± 3.93% (*p* < 0.01 *vs.* the control group). However, the treatment of different concentrations of isoquercitrin significantly attenuated H_2_O_2_-induced apoptosis from 47.09% ± 3.93% to 17.60% ± 1.15% in EA.hy926 cells. These results showed that isoquercitrin exhibited inhibitory effects on H_2_O_2_-induced EA.hy926 cell apoptosis.

**Figure 3:**
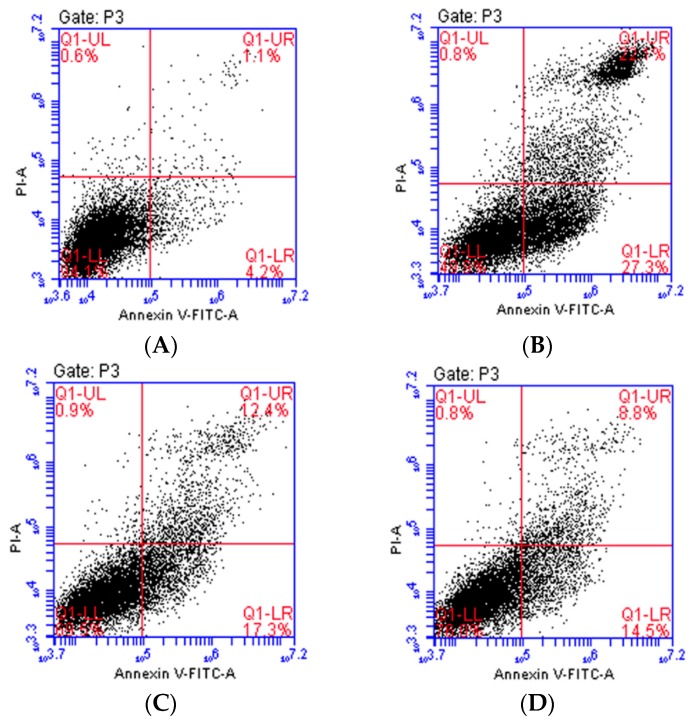

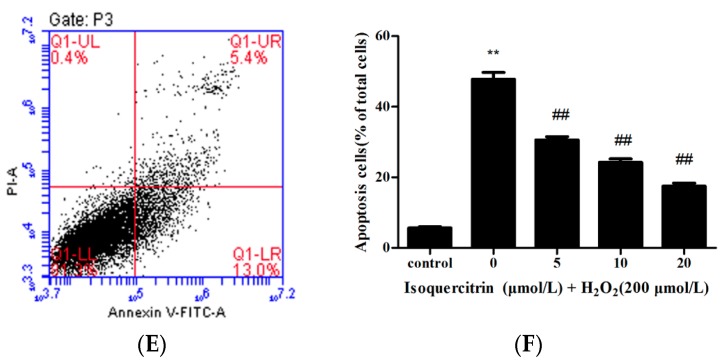
Effects of isoquercitrin on H_2_O_2_-induced apoptosis in EA.hy926 cells measured by flow cytometry. (**A**) Control group; (**B**) H_2_O_2_ treatment group; (**C**–**E**) 5, 10, and 20 μmol/L isoquercitrin, respectively, followed by the treatment of 200 μmol/L H_2_O_2_; (**F**) isoquercitrin decreased the percent of apoptotic cells induced by 200 μmol/L H_2_O_2_ in a dose-dependant manner. Data are presented as the mean ± SD (*n* = 3); ** *p* < 0.01 *vs.* the control; ^##^ *p*<0.01 *vs.* the H_2_O_2_-treated group.

To further evaluate the inhibitory effect of isoquercitrin on H_2_O_2_-induced apoptosis in EA.hy926 cells, apoptotic rates were measured by Annexin V-FITC/PI double staining using flow cytometry. As shown in Figure 3, the percentage of apoptotic cells was 5.65% ± 0.35% in the control group, whereas that of the group treated with H_2_O_2_ alone was 47.75% ± 1.95% (*p* < 0.01, *vs.* the control group). However, the treatment with 5, 10 or 20 μmol/L isoquercitrin significantly decreased apoptotic rates to 30.60% ± 0.90%, 24.45% ± 0.95%, and 17.55% ± 0.85%, respectively (*p* < 0.01, *vs.* the H_2_O_2_-treated group), in EA.hy926 cells treated with H_2_O_2_. These results suggested that isoquercitrin exhibited inhibitory effects on H_2_O_2_-induced apoptosis in EA.hy926 cells.

### 2.3. Isoquercitrin Inhibits H_2_O_2_-Induced Decreases in Mitochondrial Membrane Potential in EA.hy926 Cells

**Figure 4:**
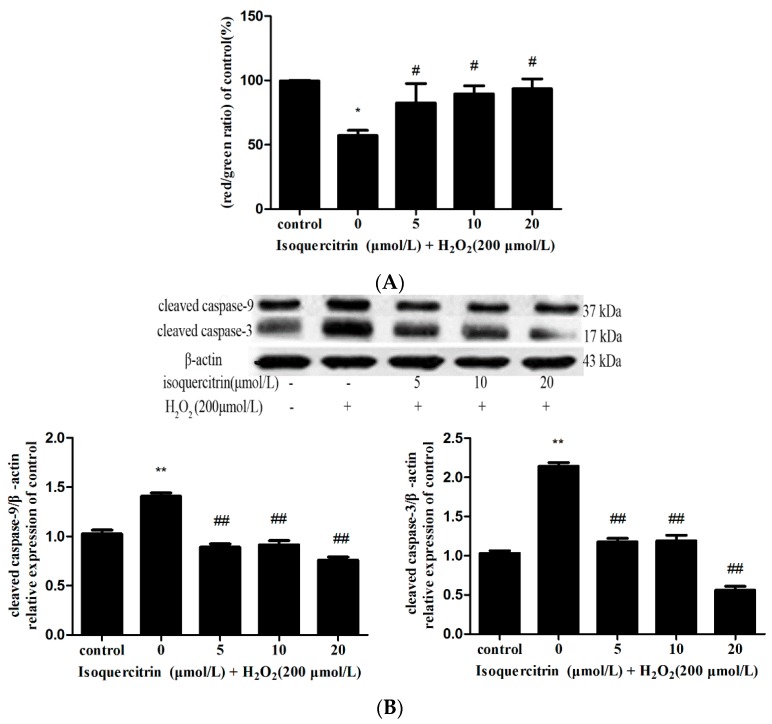

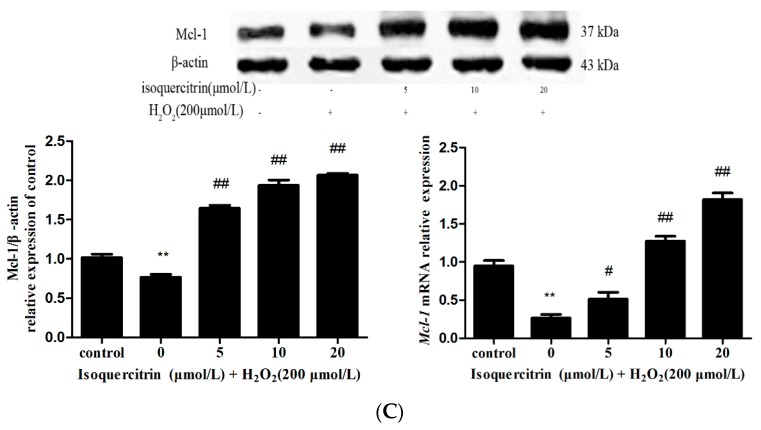
Effects of isoquercitrin on mitochondrial membrane potential and expression of cleaved caspase-3,-9 and Mcl-1 in H_2_O_2_-induced EA.hy926 cells.(**A**) Effects of isoquercitrin on mitochondrial membrane potential in H_2_O_2_-induced EA.hy926 cells; (**B**) Effects of isoquercitrin on the expression of cleaved caspase-3, cleaved caspase-9 in H_2_O_2_-induced EA.hy926 cells; (**C**) Effects of isoquercitrin on the expression of Mcl-1 in H_2_O_2_-induced EA.hy926 cells. * *p* < 0.05, ** *p* < 0.01 *vs.* the control; ^#^ *p* < 0.05, ^##^ *p* < 0.01 *vs.* the H_2_O_2_-treated group.

Mitochondrial membrane potential was assessed with JC-1 dye, a cationic lipophilic dye widely utilized in apoptosis studies using flow cytometry. As shown in Figure 4A, the ratio of red to green fluorescence intensity was significantly decreased in the group treated with H_2_O_2_ alone in comparison with that of the control group (*p* < 0.01). However, the groups pretreated with 5, 10 or 20 μmol/L isoquercitrin showed a significantly increased ratio of red/green fluorescence intensity in comparison with that of the group treated with H_2_O_2_ alone (*p* < 0.05). These results suggested that isoquercitrin inhibited H_2_O_2_-induced early apoptosis in EA.hy926 cells.

### 2.4. Isoquercitrin Inhibited H_2_O_2_-Induced Increases in Cleaved Caspase-3 and Cleaved Caspase-9 Expression in EA.hy926 Cells

In response to mitochondrial membrane injury induced by apoptosis, cytochrome c is released from mitochondria to the cytosol and binds to Apaf-1, which result in Apaf-1/cytochrome c complex formation and subsequent activation of caspase-9. Activated caspase-9 then initiates processing of caspase-3 as well as caspase-7. Activated caspase-3 in turn activates caspases-2 and -6 and also appears to be capable of acting in a feedback loop on caspase-9 to ensure complete activation of the latter. Activated caspase-6 was found to be required for the activation of caspases-8 and -10 [24,25]. Therefore, caspase-9 is key apoptosis initiator and caspase-3 is significant apoptosis executioner and the cleaved form of caspase-9, -3 can serve as a biomarker for the existence of cell apoptosis. Then, we investigated the effect of isoquercitrin on caspase-9 and caspase-3 activity by detecting the expression of cleaved caspase-9, -3 in EA.hy926 cells using western blotting. As shown in Figure 4B, H_2_O_2_ treatment significantly increased cleavage of caspase-3 and caspase-9 in comparison with that of the control group (*p* < 0.01). In contrast, pretreatment with isoquercitrin significantly decreased the expression of cleaved caspase-3 and caspase-9 in a dose-dependent manner compared with that of cells treated with H_2_O_2_ only (*p* < 0.01 *vs.* the H_2_O_2_-treated group). These results showed that isoquercitrin inhibited H_2_O_2_-induced apoptosis in EA.hy926 cells by decreasing activation of caspase-3 and caspase-9.

### 2.5. Isoquercitrin Enhanced Mcl-1 Expression in H_2_O_2_-Induced EA.hy926 Cells

Mcl-1 appears to function at an apical step in many regulatory programs that control cell survival and death which can associate with Bcl-2 family pro-apoptosis (Bax or Bak) control cytochrome c release from mitochondria [26]. To evaluate the effect of isoquercitrin on Mcl-1 in EA.hy926 cells, we investigated the expression of Mcl-1 protein and mRNA. As shown in Figure 4C, the Mcl-1 protein and mRNA levels of the group treated with H_2_O_2_ were reduced in comparison with that of the control group (*p* < 0.01). However, H_2_O_2_-treated cells pretreated with isoquercitrin showed significantly increased Mcl-1 protein and mRNA expression levels in comparison with those of H_2_O_2_-treated group (*p* < 0.01). These results indicate that isoquercitrin regulated expression of Bcl-2 family anti-apoptotic protein Mcl-1.

### 2.6. Isoquercitrin Treatment Increased Mcl-1 Expression in a PI3K/Akt/GSK3β-Dependent Manner

**Figure 5:**
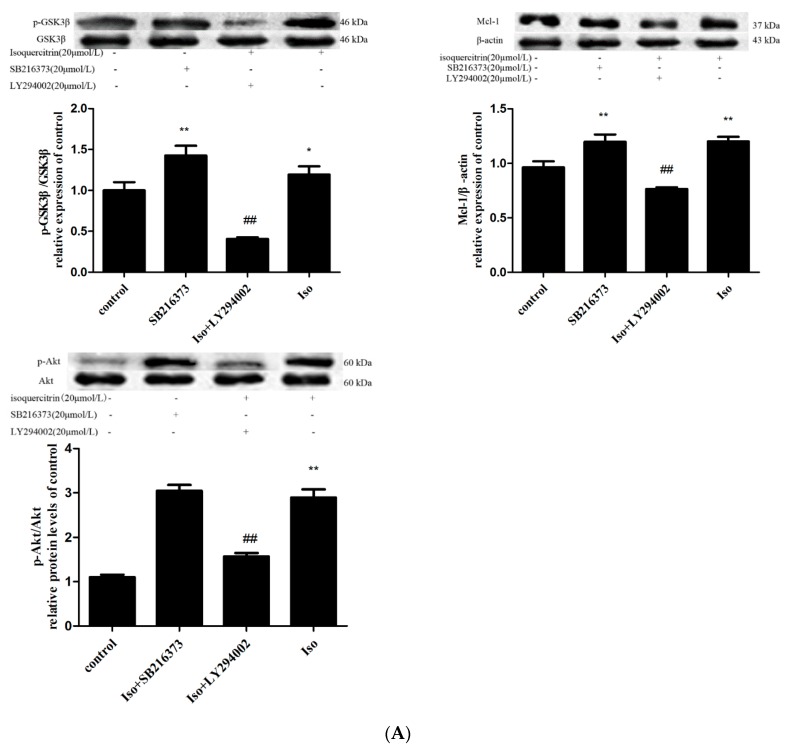

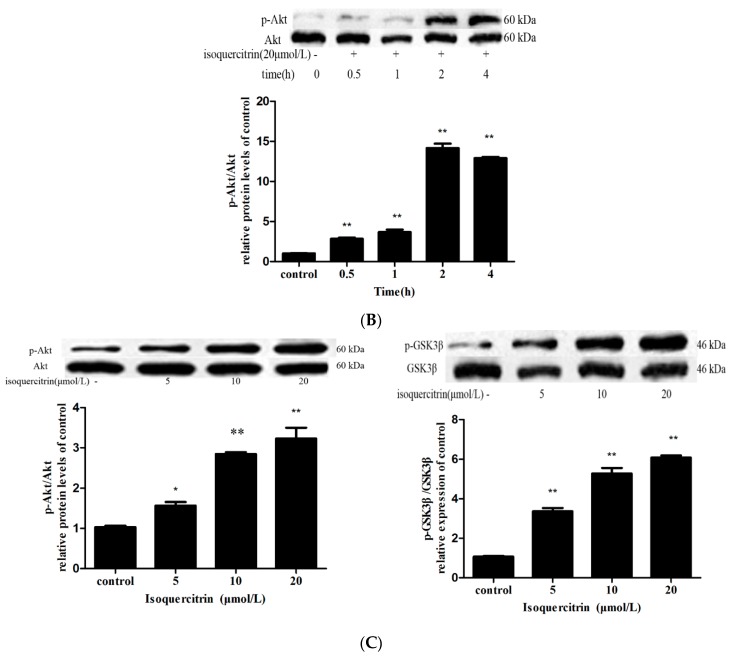
Effects of isoquercitrin on the expression of p-Akt, p-GSK3β, and Mcl-1 in EA.hy926 cells. (**A**) Effects of LY294002 and SB216373 on the expression of p-GSK3β, Mcl-1 and p-Akt in EA.hy926 cells. * *p* < 0.05, ** *p* < 0.01 *vs.* the control; ^##^ *p*< 0.01 *vs.* isoquercitrin (20 μmol/L) group; (**B**) Effects of isoquercitrin (20 μmol/L) on the expression of p-Akt in EA.hy926 cells in different time, ** *p* < 0.01 *vs.* the control; (**C**) Effects of different concentrations of isoquercitrin on the expression of p-Akt, p-GSK3β in EA.hy926 cells, ** *p* < 0.01 *vs.* the control.

Mcl-1 has a short-life, and is quickly degraded in response to a variety of apoptosis-inducing signals. GSK3β physically associated with and phosphorylated Mcl-1 and the phosphorylated Mcl-1 was then ubiquitinated and degraded by the E3 ligase β-TrCP [27]. Thus, we investigated whether GSK3β inactivation was involved in the increase in Mcl-1 expression produced by isoquercitrin treatment in EA.hy926 cells. A cell-permeable inhibitor of GSK-3β, SB216763 was used to determine if GSK3β activation is required for the reduction in Mcl-1 levels. As shown in Figure 5A, pretreatment of EA.hy926 cells with SB216763 or isoquercitrin alone increased expression levels of Mcl-1 and p-GSK3β in comparison with those of the control group (*p* < 0.01), while expression levels of Mcl-1 and p-GSK3β in EA.hy926 cells treated with isoquercitrin alone were similar to those of cells pretreated with SB216763. These data suggest that the inhibitory effect of isoquercitrin on GSK3β activation was similar to that of SB216763.

As shown above, isoquercitrin increased the stability of Mcl-1 by inactivating GSK3β. GSK3β is a ubiquitously expressed protein serine/threonine kinase, which can be inactivated by phosphorylation at Ser^9^ through a PI3K/Akt-dependent mechanism. To explore if isoquercitrin phosphorylated GSK3β through PI3K/Akt pathway. We examined phosphorylation of Akt and GSK3β by western blot analysis. First, EA.hy926 cells were incubated with 20 μmol/L isoquercitrin for 30 min, 1 h, 2 h and 4 h. As shown in Figure 5B, exposure of EA.hy926 cells to 20 μmol/L for 2 h obviously induced phosphorylation of Akt (*p* < 0.01). Therefore, EA.hy926 cells were incubated with 5, 10 and 20 μmol/L isoquercitrin for 2 h. As shown in Figure 5C, treatment of EA.hy926 cells with 5, 10, and 20 μmol/L isoquercitrin for 2 h significantly increased levels of phosphorylated Akt and GSK3β in a dose-dependent manner in comparison with those of the control group (*p* < 0.01). Additionally, cells pretreated with PI3K/Akt inhibitor LY294002 showed significantly reduced levels of GSK3β phosphorylation and Mcl-1 expression in comparison with those of cells treated with isoquercitrin (Figure 5A, *p* < 0.01). Taken together, these results indicated that induction of isoquercitrin on Mcl-1 expression is likely associated with activation of the PI3K/Akt/GSK3β signaling pathway.

## 3. Discussion

Reactive oxygen species (ROS) including superoxide anion (O^2−^), hydroxyl radical (OH^−^), and hydrogen peroxide (H_2_O_2_) are physiologically produced at a basal rate *in vivo* [28]. The cellular levels of ROS are controlled by antioxidant enzymes such as superoxide dismutase (SOD), glutathione peroxidase (GPX), catalase (CAT) and glutathione (GSH) [29]. However, different physiological or pathophysiological stimuli can generate abundant ROS which lead to oxidative stress characterized by an imbalance between the generation ROS and the capacity of the intrinsic antioxidant defense system. Oxidative stress can damage DNA, lipids, and proteins, and ultimately lead to tissue injury [30]. An increasing body of evidence suggests that oxidative stress induced by H_2_O_2_ is involved in the pathogenesis of cardiovascular diseases, such as cardiac ischemic-reperfusion injury and hypertrophy [31,32]. Therefore, inhibition of H_2_O_2_-induced oxidative stress was regarded as an important strategy for cardiovascular disease prevention.

Flavonoids are widely distributed in plants. Quercetin, one of these flavonoids, is the most abundant and widely distributed in Nature. It has been reported that quercetin has antioxidant activity by scavenging radical and preventing GSH or SOD depletion [33]. Isoquercitrin, known as quercetin-3-glucoside, also stands out among the flavonols with anti-inflammatory and antioxidant effects [34,35]. In this study, we found that isoquercitrin could inhibit oxidative stress-induced endothelial cells apoptosis. Exposure of EA.hy926 cells to 200 μmol/L H_2_O_2_ caused a decrease in cell viability and remarkably increased the apoptotic rate. However, isoquercitrin treatment attenuated the H_2_O_2_-induced decrease in cell viability and increase in apoptotic rate in a dose-dependent manner, which suggested that isoquercitrin exhibited anti-apoptotic effects in H_2_O_2_-induced EA.hy926 cells.

In addition, we explored the mechanisms underlying inhibition of H_2_O_2_-induced apoptosis by isoquercitrin. It has been defined that there is two main routes of caspase-dependent cell apoptosis. One is the death receptor-dependent pathway (extrinsic pathway) and the other is the mitochondria-dependent pathway (intrinsic pathway). The death receptor-dependent pathway triggered mainly by Fas system, for example, Fas L is able to bind to its receptor which results in the recruitment and activation of procaspase-8/-10. Caspase-8, -10, in turn, promote activation of downstream executioner caspase-3 [36]. The intrinsic mitochondrial pathway of apoptosis can be initiated by a variety of stimuli (such as radiation and free radicals), which cause changes in mitochondrial membrane and result in pro-apoptosis protein cytochrome c releasing from mitochondrial matrix to cytosol [37,38]. The intrinsic pathway was controlled by the Bcl-2 protein family, which included anti-apoptotic proteins Bcl-2, Bcl-xL, Bcl-w, Mcl-1, and A1, as well as proapoptotic proteins such as Bax, Bak, and Bok [39,40]. The hallmark of the intrinsic pathway was the release of cytochrome c into the cytosol and subsequent activation of procaspase-9 through the formation of the apoptosome, after which caspase-9 cleaved and activated effector caspase-3 [41,42]. In the present study, we found that H_2_O_2_ decreased mitochondrial membrane potential and increased expression of cleaved caspase-9 and caspase-3 in EA.hy926 cells. The effect of H_2_O_2_ was likely mediated by damage to the mitochondrial membrane, which led to release of cytochrome c and activation of procaspase-9. However, isoquercitrin pretreatment reversed this effect. Mcl-1 is a key member of the Bcl-2 family that blocks the progression of apoptosis by binding and sequestering pro-apoptotic proteins Bcl-2 Bak and Bax, which arecapable of forming pores in the mitochondrial membrane, allowing release of cytochrome c into the cytoplasm[43,44]. We explored the mechanism by which isoquercitrin inhibited H_2_O_2_-induced apoptosis via the intrinsic pathway by detecting expression of Bcl-2 family anti-apoptotic protein Mcl-1. In this study, FQ–PCR and western blotting showed that isoquercitrin significantly prevented H_2_O_2_-induced decreases in Mcl-1 mRNA and protein expression. Therefore, we speculate that isoquercitrin regulate intrinsic pathway apoptosis by inducing over expression of anti-apoptotic protein Mcl-1.

Furthermore, we explored the manner in which isoquercitrin affected expression of Mcl-1. It has been reported that Mcl-1 protein levels seem to be dynamically regulated by growth factor signaling at the protein level. Mcl-1was positively regulated by Akt and negatively regulated by GSK3β [45,46]. Phosphorylation of Mcl-1 (Ser159) by GSK3β may regulate proteasome-dependent degradation [47]. GSK-3β is a ubiquitously expressed serine/threonine protein kinase that is subject to multiple regulatory mechanisms [48,49]. The activity of GSK3β can be reduced by phosphorylation at Ser9 in a PI3K/Akt dependent manner [50,51]. To determine whether GSK3β inactivation was required for the increase in Mcl-1 expression produced by isoquercitrin treatment in EA.hy926 cells, the cells were exposed to a highly selective, cell-permeable inhibitor of GSK3β, SB216763. Expression levels of p-GSK3β and Mcl-1 were significantly increased in the SB216763-treated and isoquercitrin-treated groups in comparison with those of the control group, while there was no difference in the p-GSK3β expression levels of the isoquercitrin-treated and SB216763-treated groups. Therefore, isoquercitrin increased Mcl-1 expression by inactivating GSK3β. To further explore the manner in which isoquercitrin inactivated GSK3β, we measured phosphorylation of Akt and GSK3β. As shown in Figure 5C, isoquercitrin alone induced phosphorylation of Akt^ser473^ and GSK3β^ser9^ in a dose-dependent manner. We proposed that isoquercitrin induced inactivation of GSK3β by activating the PI3K/Akt pathway. To examine this hypothesis, LY294002 was used to block activation of the PI3K pathway. LY294002 effectively abrogated the effects of isoquercitrin on Mcl-1 expression, which indicated that the PI3K/Akt signaling pathway was involved in regulation of GSK3β phosphorylation and Mcl-1 expression in the present study.

## 4. Experimental Section

### 4.1. Materials

Isoquercitrin was purchased from the National Institutes for Food and Drug Control (Beijing, China). Dulbecco’s modified Eagle’s medium (DMEM) and fetal bovine serum (FBS) were purchased from Gibco (Grand Island, NY, USA). Antibodies against Mcl-1and β-actin were obtained from Bioworld Technology (Nanjing, China). Antibodies against Akt, p-Akt, GSK3β, p-GSK3β, cleaved caspase-3, and cleaved caspase-9 were purchased from Cell Signaling Technology (Beverly, MA, USA). Dimethyl sulfoxide (DMSO), H_2_O_2_, and 3-(4,5-dimethylthiazol-2-yl)-2,5-dephenyltetrazolium bromide (MTT) were obtained from Sigma-Aldrich Chemicals (St. Louis, MO, USA). The Annexin V-FITC apoptosis detection kit, Hoechst33342/PI apoptosis detection kit, and JC-1 detection kit were purchased from Nanjing Key Gen Biotech (Nanjing, China). All other chemicals used in the study were of analytical grade.

### 4.2. Cell Culture

The EA.hy926 cell line is an immortalized human umbilical vein endothelial cell (HUVEC) line derived from fusion of HUVECs and lung adenocarcinoma cells. EA.hy926 cells were obtained from the Shanghai Institute Cell Bank (Shanghai, China) and cultured in DMEM supplemented with 10% fetal bovine serum (FBS) and 0.1% penicillin/streptomycin in a 5% CO_2_ atmosphere at 37 °C, after which they were divided into four groups: a “control group”, an “H_2_O_2_ group”, an “isoquercitrin alone group”, and an “isoquercitrin group”. Isoquercitrin was dissolved in DMSO, which was present at a concentration less than 0.1% in all tested solutions. For all isoquercitrin group experiments, EA.hy926 cells were grown to 70%–80% confluence and treated with the designated concentrations of isoquercitrin for 24 h, after which they were exposed to 200 μmol/L H_2_O_2_ for 4 h in fresh medium and harvested for further analysis.

### 4.3. Cell Viability Analysis

MTT assays were used in the first step of determining appropriate concentrations of isoquercitrin and H_2_O_2._ EA.hy926 cells were seeded in 96-well plates (1 × 10^4^ cells/well) and cultured for 24 h. The culture medium was removed, after which the cells were pretreated with various concentrations of isoquercitrin for 24 h before exposure to H_2_O_2_ for 4 h. After the treatment, 10 μL of 5 g/L MTT solution was added to each well, after which the cells were incubated for 4 h. Next, the culture medium was replaced with 100 μL of DMSO, after which formazan crystals were dissolved by shaking the plate at room temperature. The absorbance of the solution in each well was measured at 570 nm.

### 4.4. Hoechst33342/PI Fluorescent Staining

Apoptosis was assessed using Hoechst33342/PI double fluorescent staining detection kit. EA.hy926 cells were cultured in 6-well plates at a density of 5 × 10^5^ cells per well, after which they were exposed various concentrations of isoquercitrin for 24 h, followed by exposure to 200 µmol/L H_2_O_2_ for 4 h. Next, the cells were stained with 10 µL of Hoechst 33342 solution at 37 °C for 10 min in the dark, followed by staining with 5 µL of PI at 25 °C for 15 min in the dark. The stained cells were observed under a fluorescence microscope. Cells were counted using the Image pro-plus (version 6.0.0., Media Cybernetics, Rockville, MD, USA).

### 4.5. Flow Cytometric Analysis of Apoptotic Cells

To evaluate the percentage of apoptotic cells, a double-staining assay was performed using an Annexin V-FITC/propidiumiodide(PI) kit. EA.hy926 cells were cultured in 6-well plates at a density of 5 × 10^5^ cells per well and treated with various concentrations of isoquercitrin for 24 h prior to exposure to 200 µmol/L H_2_O_2_ for 4 h. Next, the treated cells were collected, washed twice with ice-cold PBS, and resuspended in binding buffer at a concentration of 1 × 10^6^ cells/mL. Next, 5 μL of Annexin V-FITC and 5 μL of PI were added. The cells were cultured for 15 min in the dark, after which apoptotic cells were quantified using flow cytometry.

### 4.6. Mitochondrial Membrane Potential Assay

Mitochondrial membrane potential was estimated using a mitochondrial membrane potential assay kit with JC-1, a lipophilic, cationic dye that changes color as the mitochondrial membrane potential increases. EA.hy926 cells (1 × 10^4^ cells/well) were cultured in 6-well plates and treated with various concentrations of isoquercitrin for 24 h prior to exposure to 200 µmol/L H_2_O_2_ for 4 h, after which the treated cells were washed and incubated with 500 μL of JC-1 at 37 °C for 20 min in the dark. Next, the stained cells were collected, washed with JC-1 staining buffer, and resuspended in staining buffer. The fluorescence intensity of each sample was detected using flow cytometry.

### 4.7. Western Blotting

Total protein was extracted from the cells and prepared with RIPA buffer. Protein concentrations were estimated using a bicinchoninic acid protein assay kit. For western blot analysis, equal amounts of protein (40 μg) from each sample were separated and electro transferred onto NC membranes (Invitrogen, CA, USA), which were blocked in 5% nonfat milk and incubated overnight at 4 °C with primary antibodies. After three washes with Tris-buffered saline containing 0.05% Tween-20 (TBST), each membrane was incubated for 1.5 h with horseradish peroxidase (HRP)-conjugated goat anti-rabbit IgG secondary antibodies at room temperature. The bands were visualized using an ECL detection kit. Quantification of the bands was performed by densitometric analysis using Image J software (Version 1.44p, National Institutes of Health, Bethesda, MD, USA).

### 4.8. Fluorescence Quantitative PCR

Fluorescence quantitative polymerase chain reaction (FQ-PCR) was applied to determine Mcl-1 transcript levels in EA.hy926 cells. Total RNA was extracted using TRIzol reagent, after which cDNA was synthesized according to the instructions included with the PrimeScript^®^ RT reagent kit with gDNA Eraser. The reaction system was prepared according to the instructions included with the SYBR^®^ Premix Ex Taq™ kit. Following initial denaturation at 95 °C for 15 min, the amplification conditions were as follows: 45 cycles of denaturation at 95 °C for 10 s, annealing at 60 °C for 30 s, and elongation at 72 °C for 20 s. All PCR data were checked for homogeneity by dissociation curve analysis.

### 4.9. StatisticalAnalysis

Each experiment was performed at least three times. The values were expressed as the mean ± standard deviation (SD). Differences among experimental groups were evaluated by one way ANOVA. Values of *p* < 0.05 were considered statistically significant.

## 5. Conclusions

The present study provided evidences that isoquercitrin mediated antiapoptotic effects in H_2_O_2_-treated EA.hy926 cells were likely associated with inhibiting activation of caspase-3 and caspase-9, inducing Mcl-1 expression, and activating GSK3β and PI3K/Akt. These data suggested that isoquercitrin could be potential for the treatment of oxidative stress-associated cardiovascular disease.

## Abbreviations

The following abbreviations are used in this manuscript:

ROS	Reactive oxygen species
DMEM	Dulbecco’s modified Eagle’s medium
FBS	Fetal bovine serum
DMSO	Dimethyl sulfoxide
MTT	3-(4,5-Dimethylthiazol-2-yl)-2,5-dephenyltetrazolium bromide
HUVEC	Human umbilical vein endothelial cell
FBS	Fetal bovine serum
PI	Propidium iodide
TBST	Tris-buffered saline containing 0.05% Tween-20
HRP	Horseradish peroxidase
FQ-PCR	Fluorescence quantitative polymerase chain reaction
Iso	Isoquercitrin

## References

[1] Kumar P., Singh C., Agarwal N., Pandey S., Ranjan A., Singh G. (2013). Prevalence of risk factors for non-communicable disease in a rural area of Patna, Bihar—A WHO step wise approach. Indian J. Prev. Soc. Med..

[2] Străchinariu R.T. (2015). The role of endothelial dysfunction in the pathogenesis of vascular complications of diabetes mellitus—A high priority area of investigation. Romanian J. Diabetes Nutr. Metab. Dis..

[3] Rajendran P., Rengarajan T., Thangavel J., Nishigaki Y., Sakthisekaran D., Sethi G., Nishigaki I. (2013). The vascular endothelium and human diseases. Int. J. Biol. Sci..

[4] Davignon J., Ganz P. (2004). Role of endothelial dysfunction in atherosclerosis. Circulation.

[5] Anderson T.J., Uehata A., Gerhard M.D., Meredith I.T., Knab S., Delagrange D., Lieberman E.H., Ganz P., Creager M.A., Yeung A.C. (1995). Close relation of endothelial function in the human coronary and peripheral circulations. J. Am. Coll. Cardiol..

[6] Barton M., Vos I., Shaw S., Boer P., D’uscio L.V., Gröne H.J., Rabelink T.J., Lattmann T., Moreau P., Lüscher T.F. (2000). Dysfunctional renal nitric oxide synthase as a determinant of salt-sensitive hypertension mechanisms of renal artery endothelial dysfunction and role of endothelin for vascular hypertrophy and glomerulosclerosis. J. Am. Soc. Nephrol..

[7] Badran M., Ayas N., Laher I. (2014). Cardiovascular complications of sleep apnea: Role of oxidative stress. Oxid. Med. Cell. Longev..

[8] Wang D., Wang C., Wu X., Zheng W., Sandberg K., Ji H., Welch W.J., Wilcox C.S. (2014). Endothelial dysfunction and enhanced contractility in microvessels from ovariectomized rats: Roles of oxidative stress and perivascular adipose tissue. Hypertension.

[9] Sanchez-Aranguren L.C., Prada C.E., Riano-Medina C.E., Lopez M. (2014). Endothelial dysfunction and preeclampsia: Role of oxidative stress. Front. Phys..

[10] Jurikova T., Sochor J., Rop O., Mlcek J., Balla S., Szekeres L., Adam V., Kizek R. (2012). Polyphenolic profile and biological activity of Chinese hawthorn (*Crataegus pinnatifida* BUNGE) fruits. Molecules.

[11] Bhullar K.S., Rupasinghe H.P. (2015). Antioxidant and cytoprotective properties of partridgeberry polyphenols. Food Chem..

[12] Silva C.G., Raulino R.J., Cerqueira D.M., Mannarino S.C., Pereira M.D., Panek A.D., Silva J.F., Menezes F.S., Eleutherio E.C. (2009). *In vitro* and *in vivo* determination of antioxidant activity and mode of action of isoquercitrin and *Hyptis fasciculata*. Phytomed. Int. J. Phytother. Phytopharm..

[13] Williamson G., Plumb G.W., Uda Y., Price K.R., Rhodes M.J. (1996). Dietary quercetin glycosides: Antioxidant activity and induction of the anticarcinogenic phase II marker enzyme quinone reductase in Hepalclc7 cells. Carcinogenesis.

[14] Amado N.G., Cerqueira D.M., Menezes F.S., da Silva J.F., Neto V.M., Abreu J.G. (2009). Isoquercitrin isolated from *Hyptis fasciculata* reduces glioblastoma cell proliferation and changes beta-catenin cellular localization. Anti Cancer Drugs.

[15] Dos Santos A.E., Kuster R.M., Yamamoto K.A., Salles T.S., Campos R., de Meneses M.D., Soares M.R., Ferreira D. (2014). Quercetin and quercetin 3-*O*-glycosides from *Bauhinia longifolia* (Bong.) Steud. Show anti-Mayaro virus activity. Parasite Vectors.

[16] Rogerio A.P., Kanashiro A., Fontanari C., da Silva E.V., Lucisano-Valim Y.M., Soares E.G., Faccioli L.H. (2007). Anti-inflammatory activity of quercetin and isoquercitrin in experimental murine allergic asthma. Inflamm. Res..

[17] Chang L.W., Juang L.J., Wang B.S., Wang M.Y., Tai H.M., Hung W.J., Chen Y.J., Huang M.H. (2011). Antioxidant and antityrosinase activity of mulberry (*Morus alba* L.) twigs and root bark. Food Chem. Toxicol..

[18] Jung S.H., Kim B.J., Lee E.H., Osborne N.N. (2010). Isoquercitrin is the most effective antioxidant in the plant Thuja orientalis and able to counteract oxidative-induced damage to a transformed cell line (RGC-5 cells). Neurochem. Int..

[19] Gasparotto Junior A., Gasparotto F.M., Lourenco E.L., Crestani S., Stefanello M.E., Salvador M.J., da Silva-Santos J.E., Marques M.C., Kassuya C.A. (2011). Antihypertensive effects of isoquercitrin and extracts from *Tropaeolum*
*majus* L.: Evidence for the inhibition of angiotensin converting enzyme. J. Ethnopharmacol..

[20] Paulo A., Martins S., Branco P., Dias T., Borges C., Rodrigues A.I., Costa Mdo C., Teixeira A., Mota-Filipe H. (2008). The opposing effects of the flavonoids isoquercitrin and sissotrin, isolated from *Pterospartum*
*tridentatum*, on oral glucose tolerance in rats. Phytother. Res..

[21] Gasparotto Junior A., Prando T.B., LemeTdos S., Gasparotto F.M., Lourenco E.L., Rattmann Y.D., Da Silva-Santos J.E., Kassuya C.A., Marques M.C. (2012). Mechanisms underlying the diuretic effects of *Tropaeolum*
*majus* L. extracts and its main component isoquercitrin. J. Ethnopharmacol..

[22] Gasparotto Junior A., Gasparotto F.M., Boffo M.A., Lourenco E.L., Stefanello M.E., Salvador M.J., da Silva-Santos J.E., Marques M.C., Kassuya C.A. (2011). Diuretic and potassium-sparing effect of isoquercitrin—An active flavonoid of *Tropaeolum*
*majus* L.. J. Ethnopharmacol..

[23] Makino T., Kanemaru M., Okuyama S., Shimizu R., Tanaka H., Mizukami H. (2013). Anti-allergic effects of enzymatically modified isoquercitrin (α-oligoglucosyl quercetin 3-*O*-glucoside), quercetin 3-*O*-glucoside, α-oligoglucosyl rutin, and quercetin, when administered orally to mice. J. Nat. Med..

[24] Slee E.A., Harte M.T., Kluck R.M., Wolf B.B., Casiano C.A., Newmeyer D.D., Wang H.G., Reed J.C., Nicholson D.W., Alnemri E.S. (1999). Ordering the cytochrome c–initiated caspase cascade: Hierarchical activation of caspases-2,-3,-6,-7,-8, and-10 in a caspase-9—Dependent manner. J. Cell Biol..

[25] Inoue S., Browne G., Melino G., Cohen G.M. (2009). Ordering of caspases in cells undergoing apoptosis by the intrinsic pathway. Cell Death Differ..

[26] Thomas L.W., Lam C., Edwards S.W. (2010). Mcl-1; the molecular regulation of protein function. FEBS Lett..

[27] Ding Q., He X., Hsu J.M., Xia W., Chen C.T., Li L.Y., Lee D.F., Liu J.C., Zhong Q., Wang X.D., Hung M.C. (2007). Degradation of Mcl-1 by β-TrCP mediates glycogen synthase kinase 3-induced tumor suppression and chemosensitization. Mol. Cell. Biol..

[28] Hybertson B. M., Gao B., Bose S.K., McCord J.M. (2011). Oxidative stress in health and disease: The therapeutic potential of Nrf2 activation. Mol. Asp. Med..

[29] Vuleta A., Jovanović S.M., Tucić B. (2016). Adaptive flexibility of enzymatic antioxidants SOD, APX and CAT to high light stress: The clonal perennial monocot Iris pumila as a study case. Plant Physiol. Biochem..

[30] Inagi R. (2006). Oxidative stress in cardiovascular disease: A new avenue toward future therapeutic approaches. Recent Pat. Cardiovasc. Drug Discov..

[31] Slezak J., Tribulova N., Pristacova J., Uhrik B., Thomas T., Khaper N., Kaul N., Singal P. (1995). Hydrogen peroxide changes in ischemic and reperfused heart. Cytochemistry and biochemical and X-ray microanalysis. Am. J. Pathol..

[32] Chen Q.M., Tu V.C., Wu Y., Bahl J.J. (2000). Hydrogen peroxide dose dependent induction of cell death or hypertrophy in cardiomyocytes. Arch. Biochem. Biophys..

[33] Alvarez-Suarez J.M., Giampieri F., González-Paramás A.M., Damiani E., Astolfi P., Martinez-Sanchez G., Bompadre S., Quiles J.L., Santos-Buelga C., Battino M. (2012). Phenolics from monofloral honeys protect human erythrocyte membranes against oxidative damage. Food Chem. Toxicol..

[34] Valentová K., Vrba J., Bancířová M., Ulrichová J., Křen V. (2014). Isoquercitrin: Pharmacology, toxicology, and metabolism. Food Chem. Toxicol..

[35] Batista Â.G., Ferrari A.S., da Cunha D.C., da Silva J.K., Cazarin C.B.B., Correa L.C., Prado M.A., de Carvalho-Silva L.B., Esteves E.A., Júnior M.R.M. (2016). Polyphenols, antioxidants, and antimutagenic effects of Copaifera langsdorffii fruit. Food Chem..

[36] Forbes-Hernández T.Y., Giampieri F., Gasparrini M., Mazzoni L., Quiles J.L., Alvarez-Suarez J.M., Battino M. (2014). The effects of bioactive compounds from plant foods on mitochondrial function: A focus on apoptotic mechanisms. Food Chem. Toxicol..

[37] Li J., Wang Y., Du L., Xu C., Cao J., Wang Q., Liu Q., Fan F. (2014). Radiation‑induced cytochrome c release and the neuroprotective effects of the pan-caspase inhibitor z‑VAD‑fmk in the hypoglossal nucleus. Exp. Ther. Med..

[38] Jiang G.B., Zheng X., Yao J.H., Han B.J., Li W., Wang J., Huang H.L., Liu Y.J. (2014). Ruthenium(II) polypyridyl complexes induce BEL-7402 cell apoptosis by ROS-mediated mitochondrial pathway. J. Inorg. Biochem..

[39] Brunelle J.K., Letai A. (2009). Control of mitochondrial apoptosis by the Bcl-2 family. J. Cell Sci..

[40] Kuwana T., Bouchier-Hayes L., Chipuk J.E., Bonzon C., Sullivan B.A., Green D.R., Newmeyer D.D. (2005). BH3 domains of BH3-only proteins differentially regulate Bax-mediated mitochondrial membrane permeabilization both directly and indirectly. Mol. Cell.

[41] Srinivasula S.M., Ahmad M., Fernandes-Alnemri T., Alnemri E.S. (1998). Autoactivation of Procaspase-9 by Apaf-1-Mediated Oligomerization. Mol. Cell.

[42] Li P., Nijhawan D., Budihardjo I., Srinivasula S.M., Ahmad M., Alnemri E.S., Wang X. (1997). Cytochrome c and dATP-dependent formation of Apaf-1/caspase-9 complex initiates an apoptotic protease cascade. Cell.

[43] Michels J., Johnson P.W., Packham G. (2005). Mcl-1. Int. J. Biochem. Cell Biol..

[44] Willis S.N., Chen L., Dewson G., Wei A., Naik E., Fletcher J.I., Adams J.M., Huang D.C. (2005). Proapoptotic Bak is sequestered by Mcl-1 and Bcl-xL, but not Bcl-2, until displaced by BH3-only proteins. Genes Dev..

[45] Longo P.G., Laurenti L., Gobessi S., Sica S., Leone G., Efremov D.G. (2008). The Akt/Mcl-1 pathway plays a prominent role in mediating antiapoptotic signals downstream of the B-cell receptor in chronic lymphocytic leukemia B cells. Blood.

[46] Opferman J. (2006). Unraveling MCL-1 degradation. Cell Death Differ..

[47] Maurer U., Charvet C., Wagman A.S., Dejardin E., Green D.R. (2006). Glycogen synthase kinase-3 regulates mitochondrial outer membrane permeabilization and apoptosis by destabilization of MCL-1. Mol. Cell.

[48] Cohen P., Frame S. (2001). The renaissance of GSK3. Nat. Rev. Mol. Cell Biol..

[49] Maurer U., Preiss F., Brauns-Schubert P., Schlicher L., Charvet C. (2014). GSK-3—At the crossroads of cell death and survival. J. Cell Sci..

[50] Cross D.A., Alessi D.R., Cohen P., Andjelkovich M., Hemmings B.A. (1995). Inhibition of glycogen synthase kinase-3 by insulin mediated by protein kinase B. Nature.

[51] Tan J., Geng L., Yazlovitskaya E.M., Hallahan D.E. (2006). Protein kinase B/Akt-dependent phosphorylation of glycogen synthase kinase-3β in irradiated vascular endothelium. Cancer Res..

